# Factors Associated with Height Catch-Up and Catch-Down Growth Among Schoolchildren

**DOI:** 10.1371/journal.pone.0032903

**Published:** 2012-03-12

**Authors:** Rosângela F. L. Batista, Antônio A. M. Silva, Marco A. Barbieri, Vanda M. F. Simões, Heloisa Bettiol

**Affiliations:** 1 Departamento de Saúde Pública, Universidade Federal do Maranhão, São Luís, Maranhão, Brasil; 2 Departamento de Puericultura e Pediatria, Faculdade de Medicina de Ribeirão Preto, Universidade de São Paulo, Ribeirão Preto, São Paulo, Brasil; 3 Departamento de Medicina I, Universidade Federal do Maranhão, São Luís, Maranhão, Brasil; Institute of Clinical Effectiveness and Health Policy, Argentina

## Abstract

In developed countries, children with intrauterine growth restriction (IUGR) or born preterm (PT) tend to achieve catch-up growth. There is little information about height catch-up in developing countries and about height catch-down in both developed and developing countries. We studied the effect of IUGR and PT birth on height catch-up and catch-down growth of children from two cohorts of liveborn singletons. Data from 1,463 children was collected at birth and at school age in Ribeirão Preto (RP), a more developed city, and in São Luís (SL), a less developed city. A change in z-score between schoolchild height z-score and birth length z-score≥0.67 was considered catch-up; a change in z-score≤−0.67 indicated catch-down growth. The explanatory variables were: appropriate weight for gestational age/PT birth in four categories: term children without IUGR (normal), IUGR only (term with IUGR), PT only (preterm without IUGR) and preterm with IUGR; infant's sex; maternal parity, age, schooling and marital status; occupation of family head; family income and neonatal ponderal index (PI). The risk ratio for catch-up and catch-down was estimated by multinomial logistic regression for each city. In RP, preterms without IUGR (RR = 4.13) and thin children (PI<10^th^ percentile, RR = 14.39) had a higher risk of catch-down; catch-up was higher among terms with IUGR (RR = 5.53), preterms with IUGR (RR = 5.36) and children born to primiparous mothers (RR = 1.83). In SL, catch-down was higher among preterms without IUGR (RR = 5.19), girls (RR = 1.52) and children from low-income families (RR = 2.74); the lowest risk of catch-down (RR = 0.27) and the highest risk of catch-up (RR = 3.77) were observed among terms with IUGR. In both cities, terms with IUGR presented height catch-up growth whereas preterms with IUGR only had height catch-up growth in the more affluent setting. Preterms without IUGR presented height catch-down growth, suggesting that a better socioeconomic situation facilitates height catch-up and prevents height catch-down growth.

## Introduction

Catch-up and catch-down growth are defined as height or weight growth above or below the statistical limits of normality for age [1], [2]. Studies mostly conducted in developed countries have assessed the effects of intrauterine growth restriction (IUGR) and preterm (PT) birth on child growth, showing that many children achieve weight and height catch-up [3], [4], [5], [6]. Studies assessing catch-down in children who suffered IUGR or were born PT are scarce [4].

Height catch-up is mostly observed between 6 and 12 months of life in children with IUGR[7], [8] and between two and three years of age in PT children[8], [9]. The phenomenon of catch-up allows PT newborns to reach growth equivalent to that of healthy children born at term during their first years of life [10]. However, in some children (10% to 15%) height catch-up does not occur and their height may not reach its full potential [8], [11].

Variations in the compensatory growth pattern is observed in children born with IUGR, who present lower growth than adequate for gestational age (AGA) children born at term, and in PT children. In developed countries, catch-up occurs since the first six months of life and continues during infancy and adolescence, probably due to favorable socioeconomic conditions [3], [12], [13]. However, in developing countries, compensatory growth of children born with IUGR is not observed until six years of age. As a result, these children remain shorter. This indicates that their growth is chronically affected due to unfavorable intrauterine conditions. However, variations in growth velocity and final attained height occur both in developed and developing countries, as observed in Sweden [3] and Brazil [14].

There is consensus that, in both developed and developing societies, children born with IUGR continue to be smaller, to weigh less and to have lower adipose tissue growth in the first years of life than AGA or PT children [3], [12], [15], [16], [17], [18]. Despite experiencing catch-up in the first two years of life [3], [11], [19], [20], [21], babies with IUGR continue to have a relatively smaller body size in infancy than children without IUGR [22], [23], [24], [25].

The objective of the present study was to assess the effects of IUGR, PT birth and some perinatal variables on height catch-up and catch-up down growth in schoolchildren belonging to two cohorts of singleton liveborns delivered at hospitals in two Brazilian cities with contrasting socioeconomic development.

## Methods

### Study sites

The present report is part of a longitudinal, prospective study using data from two Brazilian cohorts of singleton liveborns from Ribeirão Preto, SP (1994) and São Luís, MA (1997/1998) [26]. The study sites have different socioeconomic characteristics.

Ribeirão Preto (RP), located in the Southeast of the country, a wealthier and more industrialized region with a population of 457,653 inhabitants in 1994 and 551,312 in 2005, had a Municipal-Human Development Index (M-HDI) of 0.855, occupying 22^nd^ place in the national ranking in 2000. São Luís (SL) is the capital of the State of Maranhão, located in the Northeast of Brazil, one of the poorest regions in the country with a population of 801,895 inhabitants in 1997 and 978,822 in 2005. Its M-HDI was 0.778 in 2000, corresponding to 1112nd place in the national ranking [27].

### Sampling

In RP, data were collected for all liveborns delivered during four months [28]. Hospital deliveries represented more than 99% of all births. Twin births and infants whose mothers did not reside in the city were excluded. Thus, the final sample consisted of 2,846 births. Losses represented fewer than 5% of the births.

In SL, the study was conducted from March 1997 to February 1998. A systematic sampling was performed, with proportional stratification according to the number of births at each of the 10 public and private maternity hospitals. A total of 2,541 hospital births were studied, including infants born to women residing in the city, liveborns, stillborns and single or multiple deliveries [29]. The sample was representative of the births in the city since hospital deliveries represent 96.3% of them. After multiple deliveries and stillborns were excluded, the final sample consisted of 2,443 births. Losses due to refusal or impossibility of locating the mother amounted to 5.8%.

For the follow-up at school age sampling was stratified by birth weight. Five groups were constituted: very low birth weight (VLBW, <1500 g), low birth weight (LBW, 1500 to 2499 g), insufficient birth weight (IBW, 2500 to 2999 g), and normal birth weight (NBW, 3000 to 4249 g). Children whose birth weights were at least two standard deviations above average were classified as high birth weight (HBW, ≥4250 g). All parents or persons responsible for children in the VLBW, LBW and HBW groups and one in every three in the IBW and NBW groups were invited to participate in the follow-up study. Thus, VLBW, LBW and HBW children were oversampled to increase the study power [27].

In RP, after exclusion of multiple births, stillborns and deaths during the first year of life, 1150 children were eligible for follow-up. A total of 790 children from the original birth cohort were evaluated (24 VLBW, 145 LBW, 174 IBW, 419 NBW, and 28 HBW) at age 9 to 11 years, in 2004/05, with a follow-up rate of 68.7%. In SL, after exclusion of multiple births, stillborns and deaths during the first year of life, 926 children were eligible for follow-up, with 673 children of the original cohort being followed up (5 VLBW, 76 LBW, 134 IBW, 439 NBW, and 19 HBW) at age 7 to 9 years, in 2005/06, representing a follow-up rate of 72.7%. Children whose birth length was missing were excluded. Thus, 1,402 schoolchildren remained in the analysis, 748 from RP and 654 from SL [27].

### Sample size

A sample of about 700 children in each city has an 80% power to detect a 7% difference in height catch-up or catch-down growth (estimated at about 30%) between exposed and non-exposed groups, with a 5% probability of type I error.

### Variables

The response variable was the difference between z-score for height at school age, based on the NCHS reference [30], and z-score for birth length, based on the Swedish reference [31]. It was classified using the definition proposed by Ong *et al.* (2000), who considered catch-up as a change in z-score≥0.67 and catch-down growth as a change in z-score≤−0.67.

The variable “adequate weight for gestational age/preterm birth” was created by combining preterm birth (gestational age <37 weeks, based on the date of the last normal menstrual period) and IUGR, defined on the basis of the birth weight ratio, which is the ratio between the newborn's weight and the mean weight for gestational age of the Williams *et al.* reference [32]; a birth weight ratio <0.85 was defined as IUGR [33]. This variable was categorized as follows: term newborns without IUGR (normal); preterm newborns without IUGR (preterm without IUGR); term newborns with IUGR (term with IUGR), and preterm newborns with IUGR (preterm with IUGR).

The remaining variables were: newborn's sex, maternal age (<20, 20 to 34 and ≥35 years), maternal schooling (0 to 4, 5 to 8, 9 to 11, and ≥12 years), parity (1, 2 to 4 and ≥5), maternal marital status (married, cohabiting, and no companion), family income in minimum wages (≤5, >5), and occupation of family head (non-manual, skilled and semi-skilled manual, and unskilled or unemployed). Neonatal ponderal index (PI) was calculated according to Rohrer's formula, PI = weight/length^3^×100. A child was considered “thin” when PI<10th percentile of the Lubchenco *et al.* reference [34], and “normal” otherwise.

Age groups were established as decimal ages according to the standardization recommended by Ross and Marfell-Jones [35], being 7 years for ages between 6.50 and 7.49 years, 8 years for ages between 7.50 and 8.49, and so on. A “missing” category was included for family income, because more than 10% of the families did not report income.

### Statistical analyses

Mean z-scores for birth length and schoolchild's height were calculated. Associations between preterm birth/IUGR status and z-scores for birth length and height at school age were determined by analysis of variance (ANOVA). The chi-square (χ^2^) test and, when necessary, the Fisher exact test were used to assess differences in proportions.

The risk ratios of height catch-up and catch-down growth were obtained by multinomial logistic regression in separate models for each city. Since the follow-up sampling of schoolchildren was not equiprobabilistic, because LBW, VLBW and HBW children were oversampled estimates were corrected by weighting. The variables used for weighting were birth weight and preterm birth. Sampling stratification according to birth weight was also taken into account. In the tables and in the text, absolute frequencies are presented without weighting and percentages are weighted.

### Ethics

The project was approved by the Research Ethics Committee of the Clinics Hospital, Faculty of Medicine of Ribeirão Preto, University of São Paulo (protocols 28/2004 and 10073/2009) and by the Research Ethics Committee of the University Hospital, Federal University of Maranhão (protocol 3104-476/2005). All parents or persons responsible for the children gave written informed consent to participate in the study.

## Results

In RP follow-up rates were lower among women who cohabited, who were aged <20 or from 20 to 34 years, who had ≤4 or ≥12 years of schooling, among those belonging to families whose head had an unskilled manual occupation or was unemployed and whose family income was <3 minimum wages. No differences were observed for sex or parity. In SL women with ≥12 years of schooling, primiparous women, those belonging to families whose heads were engaged in non-manual occupations and whose family income was less than three minimum wages, and boys had lower follow-up rates compared to their counterparts. There was no difference regarding maternal marital status and age (Table 1).

**Table 1:** Initial sample, eligible for follow-up, number and percentage followed-up in the 1994 Ribeirão Preto and 1997/98 São Luís birth cohorts.

Variables	Ribeirão Preto					São Luís				
	n	*n*	*n*	% followed up	p*	n	*n*	*n*	% followed up	p*
	Initial sample (excluding 48 deaths)	*Eligible for follow-up*	*followed up*			Initial sample (excluding 65 deaths)	*Eligible for follow-up*	*Followed up*		
**Occupation of household head**					0.001					<0.001
Non-manual	584	230	152	66,0		493	188	105	55,9	
Manual skilled / semiskilled	1572	643	463	72,0		1,070	419	320	76,4	
Unskilled manual / unemployed	517	221	131	59,4		750	293	225	76,8	
Missing	137	56	44	79,0		65	26	23	88,5	
**Family income (minimum wages)**					<0.001					<0.001
<3	590	251	107	42,6		1,204	473	301	63,6	
3 to 4.9	461	185	161	87,0		411	161	158	98,1	
≥5	940	369	288	78,0		606	229	169	73,8	
Missing	819	345	234	67,8		157	63	45	71,4	
**Maternal age (years)**					0.005					0.350
20 to 34	2051	832	563	67,7		1,577	610	442	72,5	
≥35	265	114	94	82,5		101	41	32	78,0	
<20	487	202	131	64,9		698	274	199	72,6	
Missing	7	2	2	85,7		2	1	0	0.0	
**Marital status**					<0.001					0.670
Married	1,664	666	489	73,5		695	266	199	74,8	
Cohabiting	690	291	158	54,2		1,107	437	314	71,9	
Single	338	147	106	72,1		575	223	160	71,7	
Missing	118	46	37	80.4		1	0	0		
**Maternal schooling (years)**					<0.001					<0.001
≥12	367	145	93	64,3		119	46	14	30.4	
9 to 11	607	246	170	69,2		841	324	255	78,7	
5 to 8	1,028	416	304	73,1		1007	397	301	75,8	
≤4	618	267	158	59,1		405	157	103	65,6	
Missing	190	76	65	85,2		6	2	0	0.0	
**Parity**					0.361					0.049
1	1148	467	313	67,0		1156	457	316	69,1	
2 to 4	1474	600	423	70.5		1119	424	321	75,7	
≥5	160	73	46	63,0		103	45	36	80.0	
Missing	28	10	8	80.0		-	-	-		
**Sex**					0.714					0.001
Male	1425	581	402	69,2		1295	509	348	68,4	
Female	1384	569	388	68,2		1083	417	325	77,9	
Missing	1	0	-			-				
**Total**	2810	1150	790	68,7		2378	926	673	72,7	

* P value calculated by the chi-square test.

In both cities, children born with IUGR had the lowest mean z-scores for birth length and schoolchild's height. The highest values for birth length were observed for preterm children without IUGR and for height at school age for normal children (terms without IUGR) in both cities. In RP, only among preterm children without IUGR mean z-score between birth and school age was reduced (from 0.25 to −0.47), whereas in SL, both normal (from −0.38 to −0.78) and preterm children without IUGR (from 0.73 to −0.97) presented reduction (Table 2).

**Table 2:** Mean z-score for birth length and for height at school age and difference between these means by preterm birth/IUGR status. Ribeirão Preto, 1994/2004–05 and São Luís, 1997–98/2005–06.

Z score	Ribeirão Preto						
		Birth length		Schoolchild's height		Z difference*	
	N	mean	95% CI**	mean	95% CI*	mean	95% CI**
**Preterm Birth/IUGR** ***							
Normal	461	−0.39	(−0.47 ; −0.31)	−0.25	(−0.32 ; −0.19)	0.13	(0.04 ; 0.22)
Preterm without IUGR	117	0.25	(0.08 ; 0.41)	−0.47	(−0.61 ; −0.33)	−0.73	(−0.93 ; −0.52)
Term with IUGR	120	−1.81	(−1.95 ; −1.66)	−0.55	(−0.71 ; −0.40)	1.25	(1.06 ; 1.44)
Preterm with IUGR	50	−1.87	(−2.09 ; −1.65)	−0.99	(−1.19 ; −0.79)	0.88	(0.62 ; 1.14)
Total	748	−0.57	(−0.64 ; −0.50)	−0.33	(−0.39; −0.27)	0.24	(0.16 ; 0.31)
p value****			p<0.001		p<0.001		p<0.001

	São Luís						
**Preterm Birth/IUGR** ***							
Normal	457	−0.38	(−0.47 ; −0.29)	−0.78	(−0.86 ; −0.70)	−0.39	(−0.50 ; −0.28)
Preterm without IUGR	74	0.73	(0.50; 0.97)	−0.97	(−1.15 ; −0.78)	−1.70	(−2.00 ; −1.41)
Term with IUGR	112	−1.77	(−1.96 ; −1.57)	−1.09	(−1.24 ; −0.94)	0.67	(0.44 ; 0.90)
Preterm with IUGR	11	−1.54	(−1.96 ; −1.12)	−1.09	(−1.56 ; −0.62)	0.44	(−0.17 ; 1.06)
Total	654	−0.49	(−0.58 ; −0.40)	−0.85	(−0.91 ; −0.78)	−0.35	(−0.45 ; −0.25)
p value****			p<0.001		p<0.001		p<0.001

* difference between birth length z-score and height at school age;

** CI – confidence interval;

*** normal: term children without IUGR – intrauterine growth restriction;

**** Calculated by ANOVA.

RP children showed higher percentage of height catch-up (32.7%; n = 255) than catch-down growth (19.3%; n = 162), whereas the contrary occurred in SL, with 21.9% (n = 154) showing height catch-up and 41.8% (n = 267) showing catch-down growth (p = 0.004).

In the non-adjusted analysis, in RP, only preterm children born without IUGR had a higher risk for height catch-down growth (RR = 3.82) at school age. Term children with IUGR and preterm children with IUGR had a risk about five-fold higher for height catch-up growth compared to normal children. Children with PI<10th percentile showed higher risk of height catch-down growth (RR = 7.26) and also of higher catch-up growth (RR = 3.65). Girls had a higher risk for catch-down (RR = 1.66) than boys. Children born to primiparous mothers had a higher risk for height catch-up (RR = 1.97) than children born to mothers who had given birth 2 to 4 times. Children born to mothers aged <20 years presented higher risk of catch-up growth (1.62) than their peers. The remaining variables were not associated with change in height z-score (Table 3).

**Table 3:** Frequency (weighted percentage) and non-adjusted risk ratio for the change in height z-score (catch-up and catch-down growth) according to birth variables. Ribeirão Preto, 1994/2004–05.

	Ribeirão Preto					
Variable	Catch-down	Catch-up	Catch-down		Catch-up	
	n (%)	n (%)	RR*	95% CI**	RR*	95% CI**
**Preterm birth/IUGR status**						
Normal***	96 (19.1)	119 (26.8)				
Preterm without IUGR	59 (51.2)	14 (10.8)	3.82	(2.36 ; 6.16)	0.57	(0.28 ; 1.13)
Term with IUGR	4 (4.0)	87 (70.4)	0.45	(0.14 ; 1.40)	5.54	(3.32 ; 9.25)
Preterm with IUGR	3 (6.5)	35 (67.4)	0.70	(0.19 ; 2.59)	5.20	(2.58 ; 10.45)
	p value<0.001****					
**Newborn's sex**						
Male	68 (15.4)	131 (34.3)				
Female	94 (23.2)	124 (31.6)	1.66	(1.09 ; 2.51)	0.99	(0.70 ; 1.42)
	p value = 0.040****					
**Neonatal ponderal index**						
Normal	155 (18,9)	246 (32.6)				
Thin (<10th percentile)	7 (15.9)	9 (45.0)	7.26	(1.93; 27.30)	3.65	(1.03; 12.89)
	p value = 0.009****					
**Maternal marital status**						
Married	105 (20.1)	152 (31.0)				
Cohabiting	37 (20.8)	51 (33.1)	1.09	(0.65 ; 1.82)	1.13	(0.71 ; 1.79)
Single	15 (13.2)	44 (38.8)	0.66	(0.33 ; 1.32)	1.27	(0.76 ; 2.11)
	p value = 0.464****					
**Parity**						
2 to 4	91 (20.1)	121 (27.5)				
1	56 (16.0)	125 (42.6)	1.01	(0.64 ; 1.57)	1.97	(1.36 ; 2.85)
≥5	14 (32.6)	9 (18.1)	1.72	(0.79 ; 3.73)	0.70	(0.28 ; 1.75)
	p value<0.001****					
**Family income (minimum wages)**						
>5	57 (22.5)	81 (34.7)				
≤5	63 (17.4)	102 (31.0)	0.64	(0.39 ; 1.03)	0.74	(0.48 ; 1.13)
Missing	42 (18.3)	72 (32.9)	0.71	(0.41 ; 1.21)	0.83	(0.52 ; 1.31)
	p value = 0.394****					
**Occupation of household head**						
Non-manual	34 (20.4)	49 (35.8)				
Manual skilled/semiskilled	100 (20.2)	147 (30.9)	0.88	(0.52 ; 1.50)	0.77	(0.48 ; 1.22)
Manual unskilled and unemployed	24 (15.6)	48 (35.2)	0.68	(0.33 ; 1.38)	0.87	(0.48 ; 1.56)
	p value = 0.634****					
**Maternal age (years)**						
20 to 34	116 (19.8)	171 (30.7)				
≥35	17 (18.1)	33 (33.3)	0.93	(0.48 ; 1.83)	1.10	(0.63 ; 1.91)
<20 years	29 (17.9)	51 (41.2)	1.09	(0.61 ; 1.92)	1.62	(1.01 ; 2.61)
	p value = 0.324****					
**Maternal schooling (years)**						
≥12	24 (25.6)	23 (27.7)				
9 to 11	35 (17.9)	59 (36.4)	0.71	(0.35 ; 1.46)	1.34	(0.68 ; 2.61)
5 to 8	56 (17.4)	106 (35.9)	0.68	(0.35 ; 1.29)	1.29	(0.70 ; 2.39)
0 to 4	35 (21.2)	48 (27.6)	0.75	(0.37 ; 1.52)	0.90	(0.46 ; 1.79)
	p value = 0.439****					

* RR- risk ratio;

** CI – confidence interval;

*** normal: term children without IUGR – intrauterine growth restriction;

**** p value excluding missing data and calculated by the chi-square test.

In the non-adjusted analysis, in SL, preterm children without IUGR had a higher risk of height catch-down (RR = 4.57) at school age, but term children with IUGR presented a lower catch-down risk (RR = 0.39) compared to normal children. In addition, term children with IUGR had a higher risk of height catch-up growth (RR = 3.28) compared to reference values. A higher risk of height catch-down was also observed for girls (RR = 1.51), for those whose mothers had <5 years of schooling (RR = 5.14) or have given birth to ≥5 children (RR = 2.71) and for children whose family income was ≤5 minimum wages (RR = 2.48). The remaining variables were not associated with change in height z-score (Table 4).

**Table 4:** Frequency (weighted percentage) and non-adjusted risk ratio for the change in height z-score (catch-up and catch-down growth) according to birth variables. São Luís, 1997–98/2005–06.

	São Luís					
Variable	Catch-down	Catch-up	Catch-down		Catch-up	
	n (%)	n (%)	RR*	95% CI**	RR*	95% CI**
**IUGR/Preterm birth status**						
Normal***	200 (42.6)	81 (18.1)				
Preterm without IUGR	52 (79.5)	6 (4.5)	4.57	(2.23 ; 9.31)	0.61	(0.16 ; 2.21)
Term with IUGR	14 (14.6)	62 (51.4)	0.39	(0.19 ; 0.79)	3.28	(1.94 ; 5.55)
Preterm with IUGR	1 (9.4)	5 (47.0)	0.19	(0.22 ; 1.75)	2.33	(0.64 ; 8.45)
	p value<0.001****					
**Newborn's sex**						
Male	129 (37.7)	81 (22.3)				
Female	138 (46.2)	73 (21.5)	1.51	(1.05 ; 2.18)	1.19	(0.77 ; 1.84)
	p value = 0.077****					
**Neonatal ponderal index**						
Normal	254 (41.7)	142 (21.9)				
Thin (<10th percentile)	13 (43.4)	12 (21.5)	1.07	(0.44; 2.56)	1.01	(0.39; 2.60)
	p value = 0.982****					
**Maternal marital status**						
Married	75 (39.5)	57 (27.6)				
Cohabiting	122 (40.2)	66 (20.3)	0.84	(0.54 ; 1.31)	0.61	(0.37 ; 1.00)
Single	70 (47.8)	31 (17.9)	1.15	(0.70 ; 1.91)	0.62	(0.33 ; 1.13)
	p value = 0.125****					
**Parity**						
2 to 4	135 (43.3)	66 (20.3)				
1	108 (37.8)	83 (24.3)	0.83	(0.57 ; 1.21)	1.14	(0.73 ; 1.77)
≥5	24 (64.2)	5 (16.0)	2.71	(1.05 ; 6.99)	1.44	(0.41 ; 5.07)
	p value = 0.068****					
**Family income (minimum wages)**						
>5	31 (25.5)	41 (29.7)				
≤5	222 (46.7)	106 (20.4)	2.48	(1.49 ; 4.13)	0.93	(0.56 ; 1.56)
Missing	14 (33.7)	7 (17.0)	1.19	(0.52 ; 2.72)	0.52	(0.19 ; 1.36)
	p value<0.001****					
**Occupation of household head**						
Non-manual	42 (41.0)	26 (24.3)				
Manual skilled/semiskilled	131 (42.8)	71 (20.2)	0.97	(0.57 ; 1.67)	0.77	(0.41 ; 1.45)
Manual unskilled and unemployed	86 (40.7)	54 (24.1)	0.97	(0.55 ; 1.72)	0.97	(0.51 ; 1.87)
	p.value = 0.854****					
**Maternal age (years)**						
20 to 34	169 (40.4)	105 (22.8)				
≥35	17 (51.6)	8 (25.7)	2.06	(0.80 ; 5.31)	1.82	(0.63 ; 1.91)
<20	81 (43.6)	41 (19.3)	1.07	(0.71 ; 1.60)	0.84	(0.51 ; 1.37)
	p value = 0.506****					
**Maternal schooling (years)**						
≥12	3 (16.8)	4 (31.9)				
9 to 11	86 (36.3)	71 (26.5)	2.99	(0.64 ; 13.91)	1.14	(0.29 ; 4.50)
5 to 8	128 (44.2)	59 (18.8)	3.65	(0.78 ; 16.89)	0.81	(0.20 ; 3.22)
0 to 4	50 (50.9)	20 (18.8)	5.14	(1.06 ; 24.88)	1.00	(0.23 ; 4.30)
	p value = 0.066****					

* RR- risk ratio;

** CI – confidence interval;

*** normal: term children without IUGR – intrauterine growth restriction;

**** p value excluding missing data and calculated by the chi-square test.

In the adjusted analysis, in RP, preterm children without IUGR (RR = 4.13) and thin children at birth (RR = 14.39) had a higher risk of height catch-down; the highest risks of height catch-up were detected in term children with IUGR (RR = 5.53), preterm children with IUGR (RR = 5.36) and children whose mothers were primiparous (RR = 1.83); PI was no longer associated with height catch-up (Table 5).

**Table 5:** Adjusted risk ratio for the changes in z-score for height (catch-up and catch-down growth) according to birth variables. Ribeirão Preto, 1994/2004–05.

	Ribeirão Preto			
Variable	Catch-down		Catch-up	
	RR*	95% CI**	RR*	95% CI**
**IUGR/Preterm birth status**				
Normal***	Reference		Reference	
Preterm without IUGR	4.13	(2.44 ; 7.00)	0.52	(0.25 ; 1.08)
Term with IUGR	0.26	(0.06 ; 1.14)	5.53	(3.13 ; 9.76)
Preterm with IUGR	0.53	(0.11 ; 2.60)	5.36	(2.57 ; 11.18)
**Sex**				
Male	Reference		Reference	
Female	1.57	(0.99 ; 2.50)	0.98	(0.64 ; 1.48)
**Neonatal ponderal index**				
Normal	Reference		Reference	
Thin (<10th percentile)	14.39	(3.36 ; 61.63)	0.80	(0.20 ; 3.15)
**Maternal marital status**				
Married	Reference		Reference	
Cohabiting	1.01	(0.54 ; 1.88)	0.95	(0.53 ;1.71)
Single	0.75	(0.34 ; 1.65)	0.75	(0.39 ; 1.43)
**Parity**				
2 to 4	Reference		Reference	
1	0.81	(0.46 ; 1.41)	1.83	(1.13 ; 2.97)
≥5	1.81	(0.74 ; 4.44)	0.51	(0.17 ; 1.51)
**Family income (minimum wages)**				
>5	Reference		Reference	
≤5	0.69	(0.37 ; 1.27)	0.70	(0.41 ; 1.20)
Missing	0.80	(0.41 ; 1.56)	0.75	(0.43 ; 1.33)
**Occupation of household head**				
Non-manual	Reference		Reference	
Manual skilled/semiskilled	0.83	(0.41 ; 1.68)	0.68	(0.35 ; 1.29)
Manual unskilled and unemployed	0.98	(0.37 ; 2.57)	0.98	(0.42 ; 2.30)
**Maternal age (years)**				
20 to 34	Reference		Reference	
≥35	0.68	(0.33 ; 1.43)	1.21	(0.64 ; 2.29)
<20	1.37	(0.68 ; 2.77)	1.43	(0.77 ; 2.65)
**Maternal schooling (years)**				
≥12	Reference		Reference	
9 to 11	0.92	(0.40 ; 2.09)	1.34	(0.61 ; 2.91)
5 to 8	0.87	(0.39 ; 1.95)	1.60	(0.72 ; 3.56)
0 to 4	1.04	(0.41 ; 2.62)	1.18	(0.46 ; 2.98)

* RR- risk ratio;

** CI – confidence interval;

*** normal: term children without IUGR – intrauterine growth restriction;

In SL, a higher risk of height catch-down was observed for preterm children without IUGR (RR = 5.19), for girls (RR = 1.52), and for children whose family income was ≤5 minimum wages (RR = 2.74); the lowest risk of height catch-down (RR = 0.27) and the highest risk of height catch-up (RR = 3.77) were also observed among term children with IUGR (Table 6).

**Table 6:** Adjusted risk ratio for the changes in z-score for height (catch-up and catch-down growth) according to birth variables. São Luís, 1997–98/2005–06.

	São Luís			
Variable	Catch-down		Catch-up	
	RR*	95% CI**	RR*	95% CI**
**IUGR/Preterm birth status**				
Normal***	Reference		Reference	
Preterm without IUGR	5.19	(2.39 ; 11.25)	0.62	(0.17 ; 2.24)
Term with IUGR	0.27	(0.12 ; 0.60)	3.77	(2.13 ; 6.65)
Preterm with IUGR	0.24	(0.02 ; 2.65)	3.28	(0.68 ;15.75)
**Sex**				
Male	Reference		Reference	
Female	1.52	(1.02 ; 2.27)	1.17	(0.73 ; 1.86)
**Neontal ponderal index**				
Normal	Reference		Reference	
Thin (<10th percentile)	2.06	(0.76 ; 5.55)	0.45	(0.16 ; 1.30)
**Maternal marital status**				
Married	Reference		Reference	
Cohabiting	0.76	(0.46 ;1.25)	0.60	(0.35 ;1.03)
Single	1.39	(0.78 ; 2.49)	0.67	(0.34 ; 1.34)
**Parity**				
2 to 4	Reference		Reference	
1	0.72	(0.46 ; 1.13)	1.03	(0.61 ; 1.73)
≥5	2.27	(0.71 ; 7.18)	1.00	(0.26 ; 3.84)
**Family income (minimum wages)**				
>5	Reference		Reference	
≤5	2.74	(1.48 ; 5.06)	0.95	(0.53 ; 1.69)
Missing	1.24	(0.48 ; 3.23)	0.77	(0.26 ; 2.23)
**Occupation of household head**				
Non-manual	Reference		Reference	
Manual skilled/semiskilled	0.81	(0.43 ; 1.52)	0.87	(0.43 ; 1.75)
Manual unskilled and unemployed	0.59	(0.30 ; 1.17)	1.08	(0.50 ; 2.31)
**Maternal age (years)**				
20 to 34	Reference		Reference	
≥35	1.48	(0.42 ; 5.14)	1.72	(0.52 ; 5.66)
<20	1.03	(0.63 ; 1.67)	0.87	(0.49 ; 1.55)
**Maternal schooling (years)**				
≥12	Reference		Reference	
9 to 11	3.96	(0.58 ; 26.97)	1.25	(0.29 ; 5.36)
5 to 8	4.04	(0.58 ; 28.18)	0.96	(0.21 ; 4.29)
0 to 4	5.19	(0.71 ; 37.84)	0.95	(0.20 ; 4.49)

* RR- risk ratio;

** CI – confidence interval;

*** normal: term children without IUGR – intrauterine growth restriction;

## Discussion

### Height growth in a middle-income country from birth to 7–11 years

Children from both cities had negative mean birth length z-scores. Thus, their mean values for birth length were below the mean values of the NCHS reference, indicating that in two different settings of a middle-income country, one more and the other less affluent, children face constraints to their growth. Preterm children without IUGR were the only exception. They showed positive mean values for birth length z-scores.

From birth to 7–11 years, although some groups caught-up with their peers, they remained showing negative mean height z-scores, indicating that constraints to their growth were alleviated but persisted through infancy and childhood. Children in the more affluent setting were able to present higher catch-up growth than those in the less affluent setting. As a result, children from the more developed setting of a middle-income country, although still presenting negative mean height z-scores at school age were closer to the mean height values of the NCHS reference than children from the less developed setting.

### Risk factors for height catch-up growth

IUGR is a heterogeneous condition affecting both term and preterm newborns. However, causes, complications and prognosis of IUGR differ between term and preterm children [4], [5]. Thus it is important to look at height catch-up separately for term and preterm children with IUGR.

Itabashi *et al.* [36], in a cohort study of SGA (small for gestational age) term and preterm newborns, showed that approximately 90% of term infants had obtained height catch-up at 5 years. In our study, in both cities, term children with IUGR presented higher height catch-up growth than normal children. In addition, children from the more affluent setting seemed to present higher height catch-up growth compared to those in the less affluent city. Thus, our results were consistent in the two cities, indicating that in a middle-income country, term children with IUGR were able to present compensatory height growth and catch-up partially with normal children. However, their height mean values were still below those from their peers from developed countries at the same age.

Results were not so consistent for preterm children with IUGR. They had higher probability of height catch-up compared to normal children in RP only, the more affluent setting. With a similar definition of height catch-up, Darendeliler *et al.* [5], in Turkey, showed that SGA preterm children were smaller and lighter than AGA (adequate for gestational age) PT children, but had significant weight and height catch-up growth, reaching the same weight and height of AGA PT children at about 5 years of age. However, Brandt *et al.* [4], in Germany, showed that 54% of SGA preterm children did not show height catch-up. In addition, Itabashi *et al.* [36], in Japan, showed that only 74% of SGA preterm newborns with a gestational age of less than 32 weeks had obtained height catch-up at 5 years. The authors concluded that SGA children with less than 32 weeks of gestation are at higher risk not to achieve catch-up growth compared to SGA children with a gestational age of more than 32 weeks. In our study, PT children with IUGR were smaller at birth than PT children without IUGR when taking into account mean z-score for birth length. In RP, the more affluent city, although PT children with IUGR presented increased catch-up growth, this compensatory growth was not enough to allow them to catch up completely with their peers. In SL, the less affluent city, preterm children with IUGR did not show height catch-up growth. As a result, at school age in both cities PT children with IUGR remained smaller than normal children. Their height mean values were approximately −1 SD below those from their counterparts in developed countries at 7–11 years.

Other findings of prospective studies conducted in developed countries also indicate that, despite partial catch-up during the first years of life [3], [7], [9], children with IUGR continue to have a slightly smaller body size compared to their peers with no IUGR during infancy [23], [24]. According to Darendeliler *et al.* [5], AGA PT children with stable intrauterine growth react in a different manner after delivery and may be more sensitive to environmental factors than SGA PT children who, once freed from the intrauterine constraints to their growth, react differently, presenting an “exaggerated” catch-up, as long as they receive good care after birth. This mechanism may explain the increase in mean height z-score between birth and school age in preterm children with IUGR compared to preterm children without IUGR in RP, the more developed city. Our results suggest that term children with IUGR tend to experience height catch-up but preterm children with IUGR experience more difficulty in reaching their potential and perhaps more so in less developed settings.

Regarding parity, there is no remarkable evidence that the number of children does interfere with height catch-up or catch-down. Our data show that in RP children of primiparous mothers had higher risk of height catch-up, whereas in SL parity was not associated with catch-up growth. Guimarães *et al.* [37], observed that children whose mothers had given birth to four or more children had a 3.5 times higher chance of having short stature than children whose mothers had given birth to only one child. In the UK, infants of primiparous pregnancies were thin at birth but showed dramatic catch-up growth, and were taller than infants of non-primiparous pregnancies from 12 mo onwards. It has been shown that availability of postnatal nutrition may be affected by family size but perhaps other unknown factors may be involved to explain why first-born children presented higher catch-up growth than their counterparts [38].

### Risk factors for height catch-down growth

In the present study, preterm children without IUGR presented higher risk of catch-down in both cities. This risk was slightly higher in SL, the less developed city. Darendelier *et al.* [5] showed that some AGA PT children presented height catch-down, with a decrease in height z-score midway through childhood in relation to birth length. The authors speculated that these AGA PT children were possibly already in a process of catch-down before birth and their postnatal growth may have been the continuation of their insufficient intrauterine growth. Alternately, as already mentioned, AGA PT children with stable intrauterine growth may be more sensitive to restrictive extrauterine environmental factors than SGA PT children. The authors concluded that AGA PT children may be at risk for impaired growth and recommended that they should be monitored as carefully as SGA children.

Children who were thin at birth showed higher risk of height catch-down at school age only in RP. Thinner children usually show catch up growth in weight, especially when they are growth-restricted [2], but in our analysis PI was adjusted for preterm birth and IUGR. So, one of the reasons why these children in the more developed city may show catch-down in height at school age is possibly because they are genetically short.

Among children younger than five years, influence of environmental factors on growth is much more important than that of genetic factors. The younger a child, the more dependent and vulnerable it is regarding the environment [39]. The observation that in SL, the less developed city, there was a slightly higher risk of height catch-down for PT children without IUGR suggests that better socioeconomic conditions facilitate height catch-up and reduce the risk of height catch-down. Although in the present study z-score distributions for birth weight and, more markedly, for height at school age were shifted towards lower values in both cities, in SL this left shift was even more intense at school age. Children from families of different socioeconomic levels have a different size, on average, at all ages, and high-income groups are always ahead along the course of maturity [39], [40]. This may explain why children born from low-income families presented higher risk of catch-down only in SL. Poverty, an inadequate diet and infections during childhood underlie unfavorable socioeconomic conditions [41]. In RP, these conditions may have been partially overcome even by low-income families due to a more equitable distribution of goods and services, including health services, in this city compared to SL.

In our study, sex interfered with growth only in SL, since girls had a higher risk of height catch-down than boys. However, no association between catch-down and sex was detected in RP. Regarding catch-up, Knops *et al.* [42], when studying factors associated with weight catch-up between 5 and 10 years of age, did not find difference between boys and girls.

### Strengths and limitations

The present study permitted evaluation of height catch-up and catch-down growth, the latter being less studied, in two cities of contrasting socioeconomic conditions in a middle-income developing country. Studies of growth in developing countries are important because factors that influence growth failure during early ages seem to differ from those observed in developed countries [43]. The present investigation had the advantage of being a longitudinal, prospective population-based cohort study. The oversampling of groups more likely to present growth faltering permitted an increase in the power of detecting differences between groups. Selective losses occurred in the sample. In SL there was a selective loss of families of more privileged socioeconomic level, in contrast to what was observed in RP, where follow-up rates were higher for the better off.

Because mortality rate of preterm infants with IUGR was higher in SL than in RP [44] fewer children in this group were alive at school age to participate in the follow-up study in SL. In addition, oversampling of preterm children in SL was not as successful as in RP.

The difficulty in comparing the present results to those reported in the literature is that most studies have investigated weight catch-up and not height catch-up. In addition, different criteria have been used to assess catch-up. While some authors consider catch-up as an increase in weight or height higher than average, others consider catch-up as an increase in z-score higher than a defined cut-off point. The more traditional criterion considers catch-up as an increase in z-score for weight and height higher than 2SD [45]. The criterion proposed by Ong *et al.* [2], which was used in the present study, considers catch-up as an increase in z-score for weight or height higher than 0.67 SD.

Another difficulty is that few studies have looked at the association between PT/IUGR and height catch-down, since most studies focused on height catch-up.

We conclude that in both cities term children with IUGR presented higher height catch-up growth than normal children. Only in RP, the more developed city, children from primiparous mothers, thin children and preterm children with IUGR had a higher risk of presenting height catch-up.

In both cities, PT children without IUGR were at a higher risk of height catch-down. Only in SL, the less developed city, children from low-income families and females were at higher risk of height catch-down.
